# The association between academic stress and test anxiety in college students: The mediating role of regulatory emotional self-efficacy and the moderating role of parental expectations

**DOI:** 10.3389/fpsyg.2023.1008679

**Published:** 2023-02-07

**Authors:** Guo Zheng, Qiongzhi Zhang, Guangming Ran

**Affiliations:** ^1^Faculty of Education, Shaanxi Normal University, Xian, China; ^2^School of Education, China West Normal University, Nanchong, China

**Keywords:** academic stress, test anxiety, regulatory emotional self-efficacy, parental expectations, moderated mediation

## Abstract

Academic stress has been showed to be an important factor associated with test anxiety. However, the internal mechanism between them is still not clear. The purpose of this study was to explore whether the relationship between academic stress and test anxiety was affected by parental expectations and regulatory emotional self-efficacy. This study recruited 1,315 volunteers aged 17–25 to complete self-reports on academic stress, parental expectations, regulatory emotional self-efficacy and test anxiety. The results showed that there was a significant positive correlation between academic stress and test anxiety. Additionally, parental expectations were negatively correlated with academic stress but positively correlated with regulatory emotional self-efficacy, and regulatory emotional self-efficacy was negatively correlated with academic stress and test anxiety. The results showed that regulatory emotional self-efficacy played a mediating role in academic stress and test anxiety, and the relationship between academic stress and regulatory emotional self-efficacy was moderated by parental expectations, which indicated that parental expectations and regulatory emotional self-efficacy may play an important role in the relationship between academic stress and test anxiety.

## Introduction

Test anxiety is a kind of trait anxiety in the examination situation, and it is restricted by individual cognition, personality and other factors (von der Embse et al., 2018; Türk and Katmer, 2019). It is a psychological state, with anxiety as the basic feature, accompanied by different emotional arousal and physiological symptoms (Zhang et al., 2019; Guo et al., 2022). Test anxiety has negative effects on students’ academic levels and mental health, such as academic difficulties (Ne Eman-Haviv and Bonny-Noach, 2019) and poor academic performance (Borekci and Uyangor, 2018; Thomas et al., 2018), and the risk of anxiety and depression also increases (Leadbeater et al., 2012). At the same time, long-term test anxiety will lead to adverse consequences for individuals’ bodies, such as sleep disorders, depression, tearfulness and eating disorders. In some cases, it may even lead to self-harm and suicidal ideation (Bentley et al., 2016; Rodway et al., 2016).

### Academic stress and test anxiety

Academic stress is an important stressor in college life which stems from subjective psychological distress in many aspects of academic learning and will have a negative impact on college students’ mental health as well as the ability to complete their studies effectively (Akgun and Ciarrochi, 2003; Sun et al., 2013; Dan et al., 2021). Several recent studies have found a significant correlation between academic stress and test anxiety (Bhat, 2017; Stankovska et al., 2018; Trigueros et al., 2020). Izzati et al. (2020) hold the view that the conceptual framework of academic consists of biological and psychosocial conceptions (including cognitive conception, emotional conception, and social behavior), which indicate that academic stress could affect individuals’ physiological and psychological state. Meanwhile, according to the deficits model of test anxiety (von der Embse et al., 2018), the physiological and psychological state caused by academic stress may result in deficits in knowledge and skills that individuals who need to perform well in evaluating test situations, and influence the occurrence and severity of test anxiety. In addition, the general strain theory also suggests that individuals are exposed to a stressful environment may develop psychological distress (Agnew, 2017), and test anxiety is the typical anxiety of this kind of psychological distress in the test situation.

### The mediating role of regulatory emotional self-efficacy

According to self-efficacy theory, self-efficacy, as the core variable of individual self-belief, is an important resource and way for individuals to cope with negative events (Bandura, 1977). Regulatory emotional self-efficacy (RESE) is a kind of self-efficacy with an emotional regulatory function that refers to the degree of confidence that individuals have in regulating their own emotional state (Bandura et al., 2003), including expressing positive emotions and managing negative emotions. Self-efficacy is often related to past experiences (Bandura, 1997). Long-term academic stress will have negative effects on students, causing depression, anxiety and despair (Huang et al., 2020; Wuthrich et al., 2020; Zhang W. J. et al., 2020; Jiang et al., 2021; Zhu et al., 2021). In the face of this stress, students’ efforts to regulate their negative emotions are often ineffective (McKay et al., 2014), which eventually leads to a decline in regulatory emotional self-efficacy. The results of previous studies showed that there was a significant negative correlation between academic stress and the ability of an individual to regulate his or her emotional responses (Arslan, 2017; Ying et al., 2020). Regulatory emotional self-efficacy can reduce vulnerability to stress and strengthen endurance to negative emotions (Bandura, 1999), while stress will lead to a decrease in academic performance and cognitive skills (Eysenck et al., 2007).

Previous studies have found that self-efficacy is an endogenous factor of test anxiety (Yao et al., 2010). Based on self-efficacy theory, the main reason why individuals have negative emotions, such as anxiety, is that they think they are unable to cope with the intractable events they are facing (Tang et al., 2010). Therefore, in the face of pressure, individuals who cannot effectively regulate their negative emotions will be anxious. Previous studies have shown that low-level self-efficacy is often accompanied with test anxiety (Chen and Ye, 2009). Regulatory emotional self-efficacy, the ability to regulate one’s emotional responses to events, has an important effect on coping with stress and improving subjective well-being (Tang et al., 2010). Individuals with high regulatory emotional self-efficacy can regulate the impact of negative events in order to avoid anxiety (Wang and Zhao, 2015). In summary, academic stress may affect test anxiety through regulatory emotional self-efficacy, regulatory emotional self-efficacy may play a mediating role in academic stress and test anxiety.

### The moderating role of parental expectations

The process of which academic stress affects regulatory emotional self-efficacy may be affected by other variables. According to Finn’s expectation theory (Finn, 1972), parental expectations could interact with students’ self-expectations, and would further influence students’ performance. We speculate that parental expectations may be related to college students’ academic stress and regulatory emotional self-efficacy. Parental expectations refer to the hope and expectations from parents of several aspects in their children’s future (Briley et al., 2014; Wang and Benner, 2014), which are formed after a comprehensive measurement of individual ability, past academic performance, ideals and family situations and has an important impact on children’s academic outcomes (Wells et al., 2013; Rauscher et al., 2017; Li et al., 2019; Pinquart and Ebeling, 2020; Li and Hu, 2021). Among the resources related to students’ learning, parental expectations are one of the most important family factors (Li and Wang, 2019; Zhang Q. et al., 2020). Previous studies have suggested that the level of academic stress on students may be inflated by excessively high levels of parental expectations (Nagle and Sharma, 2018; Subramani and Venkatachalam, 2019). The high level of parental expectations will lead to an excessive involvement of parents in their children’s lives and the over control of them. In this case, students will devote more time to studying than others and have more academic stress. Moreover, high parental expectations may have a negative effect on test anxiety (Arusha and Biswas, 2020). This may be because parental expectations may give more stress and anxiety emotions to their children (Worku et al., 2020).

Ecosystem theory states that the development of individuals is largely determined by the environment in which they live (Bronfenbrenner, 1992). As an indispensable ecosystem in the process of individual growth, the family is of vital importance for children’s development. Parental expectations for children will induce parents to make more decisions in favor of their children, giving their children a better material foundation. Parents’ attitudes and behaviors are a source of information that helps children understand themselves and build self-efficacy (Shen and Li, 2020). When parents have more expectations for their children, they will give them more care and support. This emotional and psychological support can promote the child’s regulatory emotional self-efficacy (Zeng and Liao, 2019). In conclusion, parental expectations may regulate the relationship between the academic stress of their child and his or her emotional self-efficacy.

### Current study

**Figure 1:**
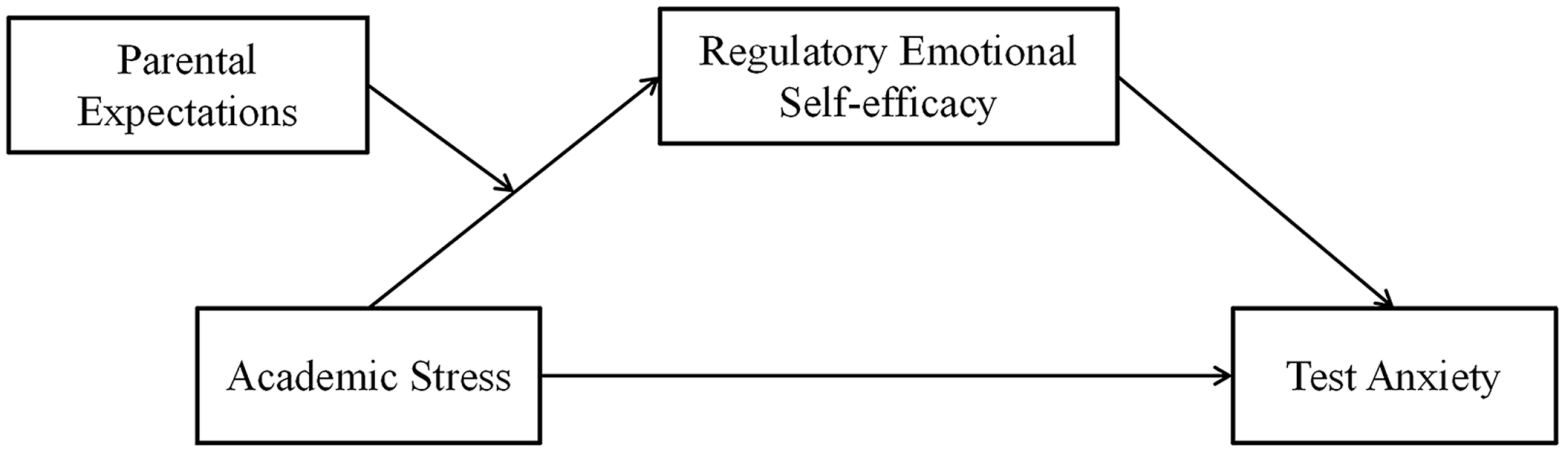
Hypothetical model of academic stress, test anxiety, parental expectations and regulatory emotional self-efficacy.

The main purpose of this study was to explore the relationship among academic stress, test anxiety, parental expectations and regulatory emotional self-efficacy. The hypothetical model is shown in Figure 1. We assumed that there was a positive correlation between academic stress and test anxiety (Hypothesis 1); parental expectations were positively correlated with both academic stress and regulatory emotional self-efficacy; regulatory emotional self-efficacy was negatively correlated with academic stress and test anxiety (Hypothesis 2); regulatory emotional self-efficacy played a mediating role in academic stress and test anxiety (Hypothesis 3); and parental expectations moderated academic stress and regulatory emotional self-efficacy (Hypothesis 4).

## Materials and methods

### Participants and procedures

A total of 1,315 participants were randomly recruited from multiple universities in Sichuan Province. The age range of the participants was 17–25 years old (*M* = 19.53, *SD* = 1.41), 576 males and 739 females; 454 only children and 861 children with siblings; 242 left-behind children and 1,073 children who lived with at least one parent.

This study was approved by the local ethics committee, and the procedure was carried out in accordance with the approved guidelines. The informed consent signed by the participants. They completed a series of questionnaires: the Chinese version of the educational stress scale for adolescents (ESSA) (Sun et al., 2011), the parental expectation questionnaire (PEQ) (Cheng, 2010), the regulatory emotional self-efficacy scale-Chinese version (RES-C) (Tang et al., 2010), and the test anxiety scale-Chinese version (TAS-CV) (Wang, 2001). The participants were asked to complete these questionnaires within 20 min.

### Measures

#### Educational stress scale for adolescents

The educational stress scale for adolescents (ESSA) compiled by Sun et al. (2011) was employed to measure the academic stress of college students. The questionnaire had a total of 16 questions in 5 dimensions: academic stress, academic burden, anxiety about performance, self-expectations and depression. Responses were measured on a 5-point scale from 1 “absolute opposition” to 5 “absolute agreement.” The higher the total score was, the greater the academic stress was. An example item was “My parents expect me to get better grades.” Cronbach’s α in this study was 0.921. CFA results showed that this scale fit the data well: *χ*^2^/df = 5.24; CFI = 0.942, TLI = 0.925; RMSEA = 0.057 (90% CI = 0.052–0.062); SRMR = 0.050.

#### Parental expectations

The parental expectation questionnaire (PEQ) was used to measure the parental expectations of the participants. The scale was compiled by Cheng (2010) and has 24 questions, covering 5 dimensions: academic achievement, future achievement, moral performance, interpersonal relationships and physical and mental quality. Responses were measured on a 5-point Likert scale from 1 “completely inconsistent” to 5 “completely consistent,” and questions 12 to 17 were reverse scored. Thus, the higher the total score was, the higher the parental expectations the participants had. An example item for this scale was “The future study and employment will bring me great stress.” Cronbach’s α in this study was 0.844. The measurement model showed a good data fit: *χ*^2^/df = 6.54; CFI = 0.914, TLI = 0.901; RMSEA = 0.065 (90% CI = 0.062–0.068); SRMR = 0.068.

#### Regulatory emotional self-efficacy

The regulatory emotional self-efficacy scale, compiled by Tang et al. (2010), was employed to measure the level of regulatory emotional self-efficacy of college students. The scale had 12 questions and was divided into three dimensions: regulating emotions in a positive emotional state, a negative or depressing episode, and an angry or furious state. Responses were measured on a 5-point Likert scale from 1 “very inconsistent” to 5 “completely consistent.” The higher the total score was, the higher the regulatory emotional self-efficacy was. An example item was “When I am alone, I can insulate myself from depression.” The scale showed good reliability and validity when verified in Chinese college students (Wu et al., 2020; Ye et al., 2021). Cronbach’s α in this study was 0.898.

#### Test anxiety

The test anxiety inventory (TAI), revised by Wang (2001), was used to measure the test anxiety level of college students. There were 37 questions in the scale, with two-point scoring used (0 represented “no,” 1 represented “yes”), among which questions 5, 26, 27, 29 and 33 were reverse scored. The higher the total score was, the higher the anxiety level. A sample item of the scale was “If I were to take an intelligence test, I would feel very anxious beforehand.” The scale has been verified to have good reliability and validity in Chinese college students (Xu et al., 2020; Huang et al., 2022). Cronbach’s α in this study was 0.861.

#### Analysis strategy

SPSS 25.0 software and the Process macro program developed by Hayes were used for data analysis, and Model 7 of 81 typical models provided by Hayes (2017) was selected for analysis.

## Results

### Common method bias inspection

In this process, an anonymous method and reverse scoring of some questions were used to control the test. The Harman single-factor test was employed to analyze common method biases. The results showed that the variation explained by the first factor was 16.70%, which was far less than the benchmark of 50% suggested by Podsakoff et al. (2003). Furthermore, there were 16 factors with characteristic roots greater than 1, which was far greater than this standard, indicating that there were no obvious common method biases in this study.

### Descriptive statistics and correlation analyses of the research variables

The means, standard deviations and gender effects of academic stress, parental expectations, regulatory emotional self-efficacy and test anxiety are shown in Table 1. The results showed that the test anxiety score of females was significantly higher than males (*p* < 0.01), and the regulatory emotional self-efficacy score of males was significantly higher than females (*p* < 0.01). The correlations between academic stress, parental expectations, regulatory emotional self-efficacy and test anxiety were listed in Table 2. Academic stress was significantly and positively correlated with test anxiety (*p* < 0.01) and negatively correlated with both parental expectations and regulatory emotional self-efficacy (*p* < 0.05, *p* < 0.01). It can be found that parental expectations were significantly and positively correlated with regulatory emotional self-efficacy (*p* < 0.01), while regulatory emotional self-efficacy was significantly and negatively correlated with test anxiety (*p* < 0.01). Additionally, gender had a significant negative correlation with regulatory emotional self-efficacy (*p* < 0.01) and a significant positive correlation with test anxiety (*p* < 0.01). Moreover, there was a significant negative correlation between age and test anxiety (*p* < 0.01). Stratification analysis was conducted to explore the relationship between academic stress and test anxiety in different grades. The results showed that there was a significant positive correlation between academic stress and test anxiety in freshmen (*r* = 0.545, *p* < 0.01), sophomore (*r* = 0.442, *p* < 0.01), junior students (*r* = 0.537, *p* < 0.01), and senior students (*r* = 0.545, *p* < 0.01).

**Table 1:** The means, standard deviations and gender effects of academic stress, parental expectations, regulatory emotional self-efficacy and test anxiety.

	Number (*N* = 1,315)		Male (*N* = 576)		Female (*N* = 739)		Gender effects	
	*M*	*SD*	*M*	*SD*	*M*	*SD*	*t*	*p*
Academic stress	51.36	10.04	50.97	10.92	51.66	9.30	−1.22	0.221
Parental expectations	82.48	10.70	83.11	12.21	81.98	9.33	1.84	0.066
Regulatory emotional self-efficacy	40.96	8.26	41.68	8.95	40.40	7.65	2.74	0.006
Test anxiety	17.46	6.99	16.89	7.31	17.91	6.70	−2.60	0.009

**Table 2:** Correlation between the variables.

	1	2	3	4	5	6
1. Gender	-					
2. Age	−0.04	-				
3. Academic stress	0.03	0.02	-			
4. Parental expectations	−0.05	−0.02	−0.07^*^	-		
5. Regulatory emotional self-efficacy	−0.08^**^	0.04	−0.44^**^	0.26^**^	-	
6. Test anxiety	0.07^**^	−0.12^**^	0.52^**^	0.02	−0.38^**^	-

^*^p < 0.05, ^**^p < 0.01.

### Academic stress and test anxiety: The moderated mediation effect test

Model 7 of the PROCESS macro program compiled by Hayes (2017) was employed to test the moderated mediation model. On the condition of controlling gender and age, taking academic stress as the independent variable, test anxiety as the dependent variable, regulatory emotional self-efficacy as the intermediary variable, and parental expectations as the adjusting variable, the bias-correct percentile *bootstrap* method was used for repeated sampling 5,000 times to test the theoretical hypothesis model (Hayes, 2017). Table 3 showed the specific results.

**Table 3:** The moderated mediation effect test.

Variable	Equation 1			Equation 2		
	Regulatory emotional self-efficacy			Test anxiety		
	*β*	*t*	95% CI	*β*	*t*	95% CI
Gender	−0.052	−2.169^*^	[−0.099, −0.005]	0.039	1.694	[−0.006, 0.084]
Age	0.056	2.363^*^	[0.010, 0.103]	−0.120	−5.217^***^	[−0.165, −0.075]
Academic stress	−0.430	−17.869^***^	[−0.477, −0.382]	0.442	17.337^***^	[0.392, 0.491]
Parental expectations	0.206	8.220^***^	[0.157, 0.255]			
Regulatory emotional self-efficacy				−0.179	−6.989^***^	[−0.229, −0.128]
Academic stress *Parental expectations	0.049	3.386^**^	[0.020, 0.077]			
*R* ^2^	0.256			0.314		
*F*	90.208^***^			149.563^***^		

^*^p < 0.05, ^**^p < 0.01, ^***^p < 0.001.

The results showed that after controlling for gender and age, academic stress significantly predicted regulatory emotional self-efficacy (*β* = −0.430, *p* < 0.001) and test anxiety (*β* = 0.442, *p* < 0.001), and regulatory emotional self-efficacy significantly predicted test anxiety (*β* = −0.179, *p* < 0.001). Based on *the bootstrap* method, it was found that regulatory emotional self-efficacy played a partially mediating role in academic stress and test anxiety (*β* = 0.077, SE = 0.013, 95%*CI* [0.050, 0.103]). In addition, the interaction between academic stress and parental expectations significantly predicted regulatory emotional self-efficacy (*β* = 0.049, *t* = 3.386, *p* < 0.01), which indicated that parental expectations could significantly moderate the impact of academic stress on regulatory emotional self-efficacy. However, the moderating effect on the relationship between academic stress and test anxiety was not significant (*β =* −0.002, SE = 0.014, 95%*CI* [−0.029, 0.025]). Moreover, parental expectations failed to moderate the association between regulatory emotional self-efficacy and test anxiety (*β =* −0.023, SE = 0.013, 95%*CI* [−0.048, 0.002]).

**Figure 2:**
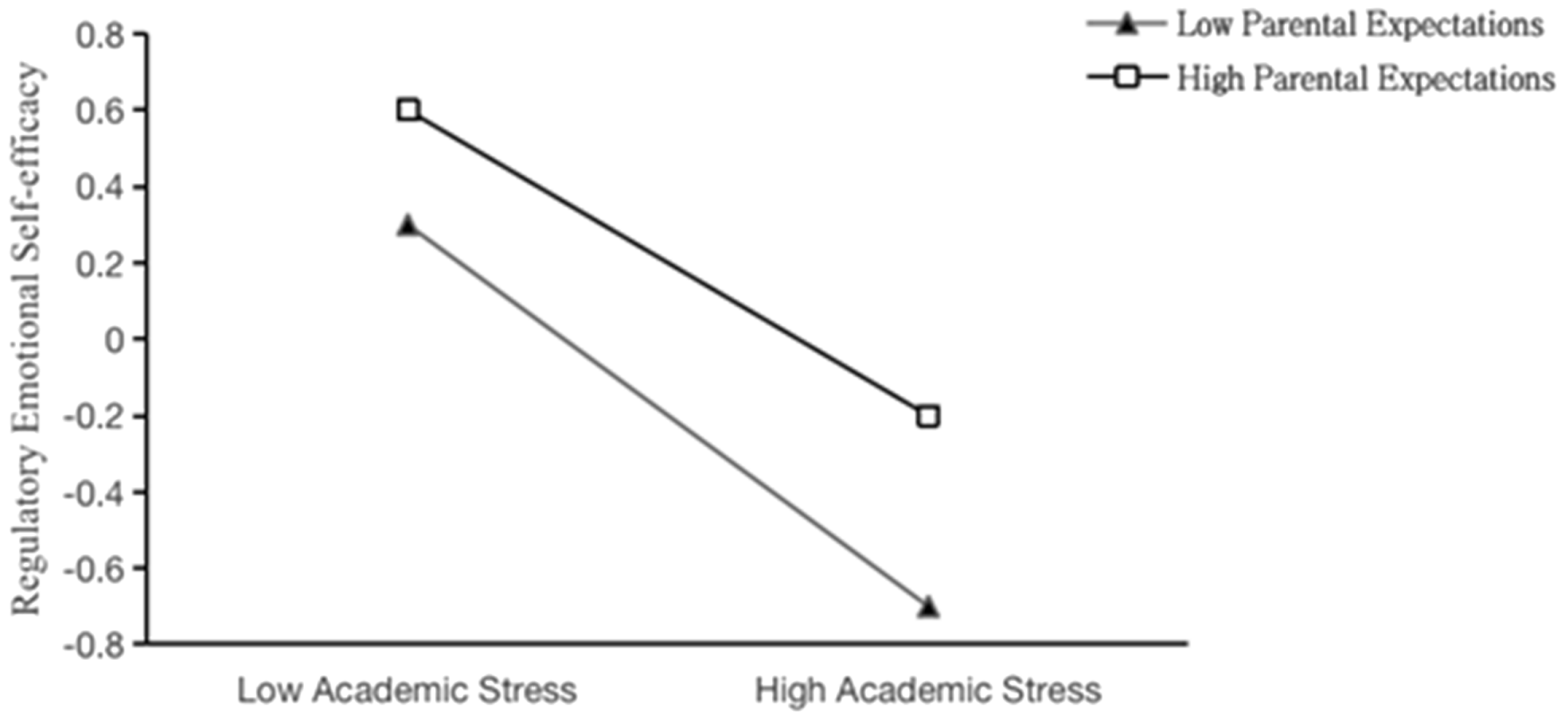
The mediating role of parental expectations in academic stress and regulatory emotional self-efficacy.

To further reveal the interaction between academic stress and parental expectations, this study conducted a simple slope test. Parental expectations were divided into high-level and low-level groups according to a standard deviation. The simple slope test was used to examine the regulation of parental expectations. The results showed that on the condition of low parental expectations (*M-SD*), academic stress had a significant negative prediction of regulatory emotional self-efficacy (*β* = −0.478, *p* < 0.001); on the condition of high parental expectations (*M + SD*), the negative prediction of academic stress on regulatory emotional self-efficacy was still significant, but the effect of prediction was weakened (*β* = −0.381, *p* < 0.001). As shown in Figure 2, with the increase in parental expectations, at a low level of parental expectations, academic stress had a stronger negative predictive effect on regulatory emotional self-efficacy.

## Discussion

This study explored the relationship between academic stress, parental expectations, regulatory emotional self-efficacy and test anxiety. The results of the correlation analysis showed that there was a significant positive correlation between academic stress and test anxiety, which verified hypothesis 1. There was a significant negative correlation between parental expectations and academic stress, a significant positive correlation between parental expectations and regulatory emotional self-efficacy, and a significant negative correlation between regulatory emotional self-efficacy and academic stress and test anxiety, which was inconsistent with hypothesis 2. Part of the relationship between academic stress and test anxiety was regulated by regulatory emotional self-efficacy, which verified hypothesis 3. More importantly, parental expectations mediated the relationship between academic stress and regulatory emotional self-efficacy, which verified hypothesis 4.

The research results showed that there was a significant difference between males and females in the test anxiety level, and the level of test anxiety in females was significantly higher than in males, which was consistent with that described in previous researches (Aydin, 2017; Sari et al., 2018; Mascret et al., 2021). In the examination situation, cognitive and psychological components were dominant for females, while behavioral or sports components were dominant for males (Wren and Benson, 2004; Aydin, 2019). Males were more inclined to regard the test as a challenge, and they would choose strategies according to their abilities to complete the task, while females were more likely to regard the test as a threatening situation, responding with fear, and found it difficult to complete the task (Torrano et al., 2020). There was a significant difference in the level of regulatory emotional self-efficacy between males and females. More specifically, the level of regulatory emotional self-efficacy of males was significantly higher than that of females, which was consistent with the results of previous studies (Calandri et al., 2021). Compared with females, males showed more confidence in dealing with negative emotions and were socially conditioned to respond with silence or positive emotions (Putwain and Daly, 2014; Wu, 2020).

This study indicated that there was a significant positive correlation between academic stress and test anxiety, that was, the higher the academic stress was, the higher the level of individual test anxiety was, which was consistent with the results of previous researches (Stankovska et al., 2018; Fuentes et al., 2019). Various courses, competitions among students, preparations for a large number of tests in a short time and employment problems created academic stress (Misra and McKean, 2000; Fang et al., 2019). Individuals who have been exposed to these kinds of pressure for a long time would increase their anxiety levels (Yeo and Lee, 2017; Zhang et al., 2022). Academic stress could not only directly affect individual test anxiety but also indirectly affect test anxiety by affecting individual cognition (Wuthrich et al., 2020).

Previous studies had rarely explored how the internal mechanism of academic stress affected test anxiety. This study found that regulatory emotional self-efficacy played a partially mediating role between academic stress and test anxiety, indicating that academic stress not only directly affected test anxiety but also affected test anxiety by reducing regulatory emotional self-efficacy. Although the beta value was small, this also suggested that regulatory emotional self-efficacy is one of the factors affecting academic stress and test anxiety. Long-term academic stress would make students feel that they were in a weak state (Muza and Muhammad, 2020), which would reduce their confidence and decrease their self-efficacy in emotion regulation. In this state, they would pay more attention to negative emotions rather than positive emotions (Huang and Guo, 2001). Besides, they could not integrate resources in time and adopt appropriate strategies to effectively deal with their own emotional problems so that they were immersed in negative emotions (Xie et al., 2022). Social cognitive theory proposed that individual self-efficacy played a key role in students’ anxiety and depression (Tahmassian and Moghadam, 2011; Wang and Liu, 2015). The stress of tests easily caused anxiety and other negative emotions. Thus, individuals who could not fully adjust their negative emotions can be overwhelmed (Tang et al., 2010), which led to an increase in test anxiety level.

It is worth noting that this study found a negative correlation between academic stress and parental expectations, which was inconsistent with results of previous researches (Nagle and Sharma, 2018). This may be because the participant group and level of parental expectations of this study were different from previous researches. More specifically, the participants in previous researches were students in middle school (Talha et al., 2020; Al Noursi and Al Daheri, 2021). Compared with middle school students, college students spent less time with their parents, and they had less needs for higher education (Högberg, 2021). As a result, parental expectations put more stress on them. In addition, previous studies suggested that high parental expectations were positively related to academic stress (Yamamoto and Holloway, 2010; Guo et al., 2020), however, the level of parental expectations in this study was not too high. This study also found that parental expectations could moderate the relationship between academic stress and regulatory emotional self-efficacy. Despite the beta value was small, this indicated that parental expectations were one of the factors influencing academic stress and regulation emotional self-efficacy. More specifically, in the condition of low level of parental expectations, academic stress had a greater negative predictive effect on regulatory emotional self-efficacy, while in the condition of a high level of parental expectations, academic stress had a smaller negative predictive effect on regulatory emotional self-efficacy. The theory of relational development systems holds the view that individual development was a two-way interaction process between individuals and the environment. Individual systems and environments could independently predict individual development and could also have an impact through their interaction (Lerner and Callina, 2013). Academic stress reflected an individual’s subjective view of academic success or failure (Buzek et al., 2019) and was a risk factor for regulatory emotional self-efficacy. Parental expectations were positive environmental factors that could reduce the negative impact of academic stress on their children’s regulatory emotional self-efficacy. At the same time, when college students perceived their parental expectations, they were motivated to enhance their achievements and self-efficacy (Zhang Q. et al., 2020). According to social capital theory (Coleman, 1988), high levels of parental expectations would affect parental investment in their children, such as providing good material conditions and investing more time and energy in parenting (Liu et al., 2020). In addition, parental expectations and attitudes would affect individuals’ values and behaviors through family socialization. Children acquired their parental expectations and formed a kind of motivational psychological energy (Goodman and Gregg, 2010). The motivational energy could help individuals cope with academic stress and improve self-efficacy. The “Rosenthal Effect” cycle model of parental expectations found that parental expectations helped improve parents’ parenting behaviors toward their children (Ma and Wei, 2017). When parental expectations was at a high level, parents would adopt more positive parenting behaviors and fewer negative parenting behaviors (Li, 2012), which will affected students’ academic attitudes and academic performance. Moreover, good parental patterns also helped students better cope with academic stress (Kour et al., 2020). Parental expectations offered children more external support to improve their academic performance and better cope with academic stress. Furthermore, a low level of parental expectations might make individuals do not take life and study seriously, resulting in poor academic performance and low self-efficacy (Ndukwu and Ndukwu, 2017; Li et al., 2020). Moreover, we found that the moderating effects on the other two pathways were not significant. This study failed to find that parental expectations were a significant predictor of test anxiety, this might be the reason why parental expectations fail to moderate the other two pathways (Wen and Ye, 2014). Parental expectations in this study included not only academic achievement, but also other aspects such as physical and mental quality (Wei and Xu, 2019; Chai et al., 2020), these aspects were not closely related to test anxiety.

### Research limitations and future directions

Researches on test anxiety have mainly focused on middle school students, and specific research on college students is not complete. This study took college students as the research participants to explore the relationship and internal mechanism between college students’ academic stress and test anxiety. However, there were still some deficiencies. First, this study was a cross-sectional study, which could not determine the causal relationship between academic stress and test anxiety. In the future, researchers can conduct longitudinal tracking or experimental designs to further explain the impact of academic stress on test anxiety. In addition, although there was no obvious common method deviation in this study, future research can collect data from multiple perspectives; for example, the expectation questionnaire for students is filled in by parents to better avoid the problems of common method biases.

## Conclusion

In summary, the present study was an attempt to explore the associations between academic stress, parental expectations, regulatory emotional self-efficacy and test anxiety. There was a significant positive correlation between academic stress and test anxiety. In addition, we found that parental expectations were negatively correlated with academic stress and positively correlated with regulatory emotional self-efficacy. Regulatory emotional self-efficacy was negatively correlated with academic stress and test anxiety. Futhermore, the relationship between academic stress and test anxiety was partially mediated by regulatory emotional self-efficacy. This indicated that regulatory emotional self-efficacy played an important role in the relation between academic stress and test anxiety. More importantly, we found that parental expectations could moderate the association between academic stress and regulatory emotional self-efficacy, suggesting that parental expectations had a significant effect on the relationship between academic stress and regulatory emotional self-efficacy. Given the limited numbers of studies examining the links among parental expectations, regulatory emotional self-efficacy, academic stress and test anxiety, further studies are needed to investigate additional factors that might link academic stress and test anxiety.

## Data availability statement

The raw data supporting the conclusions of this article will be made available by the authors, without undue reservation.

## Ethics statement

The studies involving human participants were reviewed and approved by China West Normal University. Written informed consent from the participants' legal guardian/next of kin was not required to participate in this study in accordance with the national legislation and the institutional requirements.

## Author contributions

GZ and GR contributed to the conception, design, and the examination of data of the present research. GZ and QZ were contributors to the collection and examination of data and responsible for writing. GR revised the written manuscript. All authors contributed to the article and approved the submitted version.

## Funding

This research was supported by the Meritocracy Research Funds of China West Normal University (project no. 17YC221).

## Conflict of interest

The authors declare that the research was conducted in the absence of any commercial or financial relationships that could be construed as a potential conflict of interest.

## Publisher’s note

All claims expressed in this article are solely those of the authors and do not necessarily represent those of their affiliated organizations, or those of the publisher, the editors and the reviewers. Any product that may be evaluated in this article, or claim that may be made by its manufacturer, is not guaranteed or endorsed by the publisher.
